# Is low-volume high intensity interval training a time-efficient strategy for improving body composition and cardiovascular health in children and adolescents? Evidence from a systematic review and three-level meta-analysis

**DOI:** 10.3389/fphys.2025.1736441

**Published:** 2025-12-19

**Authors:** Weihua Zheng, Yue Xing, Mingyue Yin, Yan Guo, Shunzhe Piao, Yang Cao, Hongbo Chen, Hansen Li

**Affiliations:** 1 School of Social Sports, Shenyang Sport University, Shengyang, China; 2 Australian Catholic University, East Melbourne, VIC, Australia; 3 Sport Institute, Hua Qiao University, Quanzhou, China; 4 Clinical Epidemiology and Biostatistics, School of Medical Sciences Faculty of Medicine and Health, Örebro University, Örebro, Sweden; 5 Unit of Integrative Epidemiology, Institute of Environmental Medicine, Karolinska Institutet, Stockholm, Sweden; 6 Fuzhou Software Technology Vocational College, Department of Military and Physical Education, Fuzhou, Fujian, China; 7 Universiti Malaya, Faculty of Sports and Exercise Science, Kuala Lumpur, Malaysia; 8 School of Physical Education, Sichuan Agricultural University, Ya’an, China

**Keywords:** LV-HIIT, children, adolescents, time-efficient strategy, body composition, cardiovascular health

## Abstract

**Objectives:**

This meta-analysis assessed the impact of low-volume high-intensity interval training (LV-HIIT) on body composition and cardiovascular health in children and adolescents, while examining potential moderating factors.

**Methods:**

A systematic search was conducted in PubMed, Web of Science, the Cochrane Library (CENTRAL), and CNKI from inception to April 2025. A three-level random-effects model was used to estimate the overall effects, and subgroup analyses supplemented with meta-regression were performed to explore potential moderators and sources of heterogeneity.

**Results:**

A total of 23 studies (996 participants, including 246 females) were included, with 6 studies on normal-weight and 17 on overweight/obese individuals. Compared with controls, low-volume high-intensity interval training (LV-HIIT) significantly reduced BMI (g = −1.24), fat mass (g = −0.99), body fat (g = −0.89), waistline (g = −0.42), weight (g = −0.34), and SBP (g = −0.37), while improving VO_2_max (g = 1.35). No significant differences were observed versus MICT. Subgroup and dose-response regressions suggested that weight status, age, intervention duration, training frequency, repetitions, and per-repetition time may alter the observed effects. Descriptive findings indicated comparable effects of LV-HIIT with small-sided games and sprint interval training but greater benefits over moderate-intensity interval training

**Conclusion:**

LV-HIIT can effectively and time-efficiently improve body composition and cardiovascular health in children and adolescents, with overall effects comparable to MICT. Exercise prescriptions should carefully consider weight status, age, and intervention characteristics; however, given the limited number of studies and potential bias, the conclusions should be interpreted with caution. Limited descriptive comparisons indicate that LV-HIIT produces effects similar to SSG and SIT, and may offer greater benefits than MIIT.

**Systematic Review Registration:**

https://osf.io/exhjm/.

## 1 Introduction

The World Health Organization (WHO) recommends that children and adolescents aged 5 to 17 should engage in an average of at least 60 min of moderate-to-vigorous physical activity daily, primarily aerobic exercise, and perform vigorous-intensity as well as muscle- and bone-strengthening activities at least three times per week (Chaput et al., 2020). However, numerous studies have reported that most children and adolescents fail to meet these guidelines (Aubert et al., 2021; Sluijs et al., 2021), largely due to factors such as academic pressure, increased screen time, and environmental constraints, which collectively contribute to physical inactivity (Falese et al., 2021; Silva et al., 2022). Prolonged physical inactivity is closely correlated with multiple health risks, including obesity, reduced cardiovascular health, psychological problems, and an increased likelihood of developing chronic diseases later in life (Ekelund et al., 2019). Obesity and cardiovascular health are recognized as critical indicators of youth development. Accumulating evidence indicates that obesity during childhood and adolescence not only adversely affects pubertal development but also substantially increases the risk of obesity in adulthood (Biro and Wien, 2010; Crocker et al., 2014; Simmonds et al., 2016; Ward et al., 2017), while low cardiovascular health is strongly linked to elevated body mass index, a higher prevalence of metabolic syndrome, and increased all-cause mortality risk (Mintjens et al., 2018; Haapala et al., 2022). Therefore, it is necessary to develop practical supplementary strategies to help children and adolescents accumulate physical activity throughout the day, thereby improving body composition and cardiovascular health.

Among a variety of physical activity strategies, high-intensity interval training (HIIT) has emerged as an effective exercise approach for improving health-related fitness in children and adolescents (Eddolls et al., 2017). HIIT refers to an exercise modality characterized by repeated bouts of high-intensity effort, typically corresponding to 64%–90% of VO_2_max or 77%–95% of HRmax—interspersed with periods of active or passive recovery (Gillen and Gibala, 2013). In addition, studies have shown that children and adolescents are less likely to participate in structured exercise programs solely for health purposes; however, HIIT represents a potential option that can be integrated into physical education classes and sports training (Engel et al., 2018; Poon et al., 2024). However, traditional HIIT protocols often have a total duration of 25–40 min per session (Yin et al., 2024e), which does not fully meet the criteria for time efficiency. Evidence indicates that HIIT interventions with longer durations can lead to higher dropout rates (Vollaard and Metcalfe, 2017; Vollaard et al., 2017; Reljic et al., 2022). For adolescents, both time and socioeconomic status are significant factors influencing their participation in physical activity (Dagkas and Stathi, 2007). Since schools are places where children and adolescents spend most of their time, the accumulation of physical activity can occur not only during physical education classes but also throughout various fragmented periods during the day (Costigan et al., 2015; Brazendale et al., 2017). Therefore, HIIT programs designed to align with the fragmented time structure of children’s and adolescents’ daily routines may offer a more feasible and effective approach for implementation in real-world settings.

The total duration of HIIT is closely related to its training volume. Although Sultana et al. (2019) defined low exercise volume as a weekly metabolic equivalent (MET) expenditure ≤500, other studies have proposed using high-intensity exercise duration ≤15 min as a defining criterion (Taylor et al., 2019; Sabag et al., 2022). While the MET-based approach provides quantitative estimates, it does not account for interindividual intensity differences or accurately reflect accumulated high-intensity time, limiting cross-study comparability. By contrast, defining low-volume HIIT as total duration ≤30 min with cumulative high-intensity time ≤15 min offers a more intuitive and widely used standard (Weston et al., 2014; Yin et al., 2024e; Lu et al., 2025). Within this framework, Gibala and colleagues proposed two even shorter HIIT models, including sprint interval training (SIT) (Gibala et al., 2014; Gibala and Little, 2020). The first involves a total duration ≤30 min with ≤10 min of vigorous exercise including warm-up, recovery, and cool-down, while the second is more extreme, with a total exercise duration ≤15 min encompassing all phases and no more than 5 min of vigorous effort. Given that the present study focuses on children and adolescents, developmental differences in exercise physiology must be considered. Previous research has shown that, compared with adults, children can more easily reach maximal exercise intensities and tolerate more repetitions, yet are unable to sustain each high-intensity effort for extended periods (Billat, 2001; Ratel et al., 2005; Chatzilazaridis et al., 2024). Therefore, this study adopts a conservative time threshold to identify low-volume high-intensity interval training (LV-HIIT). Interventions with less than 15 min of high-intensity exercise may also increase heterogeneity, as exposure time can vary up to threefold between studies (Buchheit and Laursen, 2013).

Since there is currently no universally accepted definition of LV-HIIT, we adopted a definition based on both time efficiency and safety considerations for children and adolescents. Following the framework proposed by Gibala et al. (2014), LV-HIIT was defined as a training protocol with a total session duration not exceeding 30 min (including warm-up, inter-bout recovery, and cool-down), of which the total vigorous exercise time does not exceed 10 min. This definition ensures that the essential characteristics of low-volume training are maintained while balancing the needs for time efficiency and exercise safety in children and adolescents.

To the best of our knowledge, existing studies have primarily compared HIIT with moderate-intensity continuous training (MICT). Current evidence suggests that HIIT is more effective than MICT in improving maximal oxygen uptake (VO_2_max) and systolic blood pressure (SBP), whereas similar effects have been observed for diastolic blood pressure (DBP) (Deng and Wang, 2024; Zheng et al., 2025). However, findings regarding body composition remain inconsistent. For example, Wang et al. (2024) reported that HIIT significantly reduced bodyweight compared with MICT, while Yin et al. (2020) found no significant differences in any anthropometric indicators. Such discrepancies may stem from the fact that these studies evaluated the aggregated effects of various LV-HIIT protocols without distinguishing between differences in training volume and other design characteristics. In other words, they overlooked the moderating role of HIIT’s intrinsic features. Although LV-HIIT involves substantially lower total training volume, brief intense metabolic stress can still induce meaningful physiological adaptations. Short bouts of high metabolic stress rapidly activate AMPK-driven signaling and upregulate PGC-1α, a key regulator of mitochondrial biogenesis, thereby enhancing oxidative capacity and metabolic efficiency (Gillen and Gibala, 2013). These mechanistic responses provide a biological rationale for why LV-HIIT may produce improvements comparable to—or in some cases greater than—those elicited by higher-volume endurance training. This observation underscores the need to independently and systematically investigate the effects of LV-HIIT on body composition and cardiovascular health in children and adolescents. Furthermore, to date, no systematic review or meta-analysis has specifically examined the effects of LV-HIIT in children and adolescent populations. The absence of such comprehensive evidence limits our understanding of the practical application and current research landscape of LV-HIIT among the general public.

In response to aforementioned research gaps, we conducted a meta-analysis to comprehensively evaluate the effects of LV-HIIT on body composition and cardiovascular health in children and adolescents, and to compare its outcomes with those of moderate-intensity continuous training (MICT) and no-exercise control groups. In addition, several included studies examined comparisons between LV-HIIT and other exercise modalities, such as moderate-intensity interval training (MIIT), small-sided games (SSG), and sprint interval training (SIT). Given the limited number of these studies, their findings were narratively summarized in the results section. Finally, subgroup analyses were performed to identify potential moderators influencing the effects, and dose–response relationships were explored to provide additional insights into the training characteristics of LV-HIIT.

## 2 Methods

This systematic review and meta-analysis was conducted in accordance with the Preferred Reporting Items for Systematic Reviews and Meta-Analyses (PRISMA) guidelines (Parums, 2021) and has been prospectively registered with the Open Science Framework (OSF; https://osf.io/crqns/).

### 2.1 Search strategy

This study systematically searched the following databases: PubMed, Web of Science, the Cochrane Library (CENTRAL), and China National Knowledge Infrastructure (CNKI). Using PubMed as an example, the search strategy consisted of three groups of keywords combined with Boolean operators “OR” and “AND.” The exercise intervention–related search string was (“high-intensity interval training” OR “sprint interval training” OR “interval training” OR “intermittent training” OR “interval exercise” OR “intermittent exercise” OR “HIIT” OR “HIIE” OR “SIT” OR “low-volume HIIT” OR “low-volume high-intensity interval training”). The population-related search string was (“child” OR “children” OR “adolescent” OR “adolescents” OR “youth” OR “teenager” OR “teenagers” OR “pediatric” OR “paediatric” OR “juvenile”). The outcome-related search string was (“BMI” OR “body mass index” OR “waist circumference” OR “hip circumference” OR “waist-to-hip ratio” OR “fat-free mass” OR “FFM” OR “resting heart rate” OR “body fat” OR “lean body mass” OR “blood pressure” OR “VO_2_max” OR “heart rate recovery” OR “CRF” OR “HRR” OR “SBP” OR “DBP”) (Yin et al., 2024e; Lu et al., 2025). The final search formula was (exercise intervention terms) AND (population terms) AND (outcome terms). Only full-text articles published in English or Chinese were included. To ensure completeness, the reference lists and forward citations of all included studies were manually screened. Detailed search terms are provided in Supplementary Datasheet S1.

### 2.2 Inclusion and exclusion criteria

The inclusion criteria were pre-defined based on the PICOS framework (Population, Intervention, Comparison, Outcomes, and Study design) (Amir-Behghadami and Janati, 2020). The search covered studies from database inception to April 2025 to ensure comprehensive identification of all potentially relevant publications, and strict eligibility criteria were applied to maintain the completeness and reliability of the results. According to the World Health Organization (WHO) 2007 Growth Reference Data for 5–19 Years (De Onis, 2007), our detailed criteria were designed as follows.

1. Population (P): participants were children (5–12 years) and adolescents (13–19 years) aged <18 years, with no medical conditions preventing engagement in physical activity or exercise.
2. Intervention (I): studies were required to include LV-HIIT, defined as training performed at an intensity of 64%–90% VO_2_max, 77%–95% HRmax, or 60%–89% HRR, or with a rating of perceived exertion (RPE) ≥14 (Coates et al., 2023). Each training session had a total duration (including warm-up, main exercise, and recovery) ≤30 min, with total high-intensity exercise time ≤10 min (Gibala et al., 2014). When the intervention was SIT, the duration of each sprint bout was ≤30 s. Intervention programs were required to last ≥2 weeks and clearly report key exercise prescription components, including frequency, mode, intensity, and training volume.

Given the substantial heterogeneity in how exercise intensity was quantified across the included studies, all extracted intensity indicators were systematically organized and classified by their physiological characteristics (Table 1) to enable a clearer and more coherent comparison. Heart rate–based measures such as %HRmax and %HRpeak, and metabolic measures such as %VO_2_max and %VO_2_peak, were retained as direct representations of relative intensity. Although metrics such as MAS, MAP, and HRR can reflect aerobic metabolic demand, they do not show a linear correspondence with intensity domains derived from VO_2_max or HRmax. This is particularly evident in adolescents, where the relationship between heart rate and metabolic rate is markedly nonlinear. For this reason, these indicators were considered only as approximate markers of metabolic intensity and were presented descriptively without numerical conversion. All-out sprint efforts and RPE values cannot be reliably standardized into physiological intensity domains and were therefore preserved as non-convertible measures. This structured but non-coercive standardization strategy allows for improved interpretability of exercise intensity across studies while minimizing error that may arise from forced physiological conversions.

3. Comparison (C): control conditions could include no-exercise controls, MICT, or any HIIT protocol not meeting the LV-HIIT definition.
4. Outcomes (O): studies were required to report at least one outcome related to cardiovascular health or body composition. (5) Study design: only randomized controlled trials (RCT) or controlled trials (CT) were included. Exclusion criteria were as follows: non-English or non-Chinese publications, qualitative studies, systematic reviews or meta-analyses, study protocols, grey literature, conference abstracts without full text, and review articles.

**TABLE 1:** Standardization of exercise intensity indicators.

Category	Metric type	Examples	Notes
HR-based	%HRmax, %HRpeak	≥77%–95% HRmax, HRpeak	Direct relative intensity
VO_2_-based	%VO_2_max, %VO_2_peak	≥64%–90% VO_2_max, VO_2_peak	Direct relative intensity
Approx.metabolic	MAS, MAP, HRR	≥100%MAS, MAP, 60%HRR	Approximate only
Non-convertible	All-out, RPE	30-s sprint; RPE ≥14	Cannot be converted

### 2.3 Study selection and data extraction

Duplicate records identified during the search were removed by an independent reviewer (ZWH) using EndNote 20 software. Subsequently, two reviewers (ZWH and XY) independently screened the remaining studies using Zotero 7 software according to the predefined inclusion and exclusion criteria. Studies that could not be excluded based solely on titles and abstracts were retrieved for full-text evaluation. Any disagreements arising during the screening process were resolved through consultation with a third reviewer (YMY).

Data extraction was performed by the same two reviewers (ZWH and XY) who participated in the screening stage, using a customized Excel extraction form developed prior to full-text screening. The reviewers independently extracted the following information: study authors and details, participant characteristics, exercise intervention specifics, and outcome measures. A third reviewer (YMY) conducted an additional round of verification. In cases of disagreement, a fourth independent reviewer (LHS) was consulted to reach consensus. When outcome data were missing or presented only in graphical form, the study authors were contacted to obtain the required information. If no response was received after the initial email, a follow-up email was sent after a 48-hour interval. Studies were excluded if no reply was obtained within 2 days, and data could not be retrieved.

### 2.4 Risk of bias and certainty of evidence

The risk of bias was assessed using the Cochrane Collaboration’s Risk of Bias 2 (RoB 2) tool, which evaluates the following domains: random sequence generation, allocation concealment, blinding of participants and personnel, blinding of outcome assessment, completeness of outcome data, selective reporting, and other potential sources of bias (Sterne et al., 2019). Two reviewers (ZWH and XY) independently performed the assessments, and any disagreements were resolved through discussion. If consensus could not be reached, a third reviewer (YMY) acted as the adjudicator.

The certainty of evidence for each outcome was evaluated using the Grading of Recommendations Assessment, Development and Evaluation (GRADE) approach, considering five domains of potential downgrading: risk of bias, inconsistency, indirectness, imprecision, and publication bias (Guyatt et al., 2008). The overall quality of evidence was classified into four levels: high, moderate, low, and very low. The grading assessment was performed by one reviewer (ZWH) and verified by a second reviewer (YMY).

### 2.5 Statistical analysis

As most included studies reported multiple outcome measures, treating these effect sizes as independent could underestimate within-study correlations and lead to biased variance estimates. Therefore, a three-level meta-analytic model was applied to account for the dependency among multiple effect sizes derived from the same study. In this framework, Level 1 represents the sampling variance of individual effect sizes, Level 2 captures the variance among different outcomes within the same study, and Level 3 reflects the variance between studies. This hierarchical modeling approach allows effect sizes from different outcomes to be appropriately clustered at the study level, thereby avoiding erroneous assumptions of independence and providing more accurate estimates of the overall effect and heterogeneity (Assink and Wibbelink, 2016; Cheung, 2019). In addition, for studies that compared LV-HIIT with other exercise modalities (moderate-intensity interval training (MIIT), small-sided games (SSG), and sprint interval training (SIT)), a narrative synthesis was conducted instead of meta-analysis due to the limited number of eligible studies. The mean change (Mchange) and standard deviation of change (SDchange) were calculated using the following formulas. The mean change and its corresponding standard deviation were calculated using Equations 1, 2, respectively:

$$M_{\text{change}}=M_{\text{post}}-M_{\text{pre}}$$ (1)

$${\;\text{SD}}_{\text{change}}=\sqrt{{\text{SD}}_{\text{pre}}^{2}+{\text{SD}}_{\text{post}}^{2}-\left(2\times r\times {\text{SD}}_{\text{pre}}\times {\text{SD}}_{\text{post}}\right)}$$ (2)

Where M_post_ and M_pre_ represent the post- and pre-intervention means for the LV-HIIT or control group, and SD_pre_ and SD_post_ denote the corresponding standard deviations. The correlation coefficient (r) refers to the relationship between pre- and post-intervention measures. As few included studies reported this correlation, a value of r = 0.50 was assumed in accordance with the recommendations of the Cochrane Handbook (Cumpston et al., 2019). To correct for small-sample bias, Hedges’ g was used as the effect size and was calculated as shown in Equation 3 (Hedges and Olkin, 1985):

$$Hedge^{\prime }s\;g=\frac{LV‐HIIT\left(M_{change}\right)-CON\left(M_{change}\right)}{SD_{pooled}}\times \left(1-\frac{3}{4\left(n_{1}+n_{2}\right)‐9}\right)$$ (3)

The formula represents the mean difference between the LV-HIIT and control groups, where n_1_ and n_2_ denote the sample sizes of each group, and the pooled standard deviation is calculated as shown in Equation 4:

$${\text{SD}}_{pooled}=\sqrt{\frac{\left(\left(n_{1}-1\right)\times {\text{SD}}_{1}^{2}+\left(n_{2}-1\right)\times {\text{SD}}_{2}^{2}\right)}{\left(n_{1}+n_{2}‐2\right)}}$$ (4)

When the standard error (SE) was reported in a study, it was converted using the following formula shown in Equation 5:

$$SD=SE\times \sqrt{N}$$ (5)

In the formula, N represents the sample size. The magnitude of the Hedges’ g effect size was interpreted as follows: trivial (<0.2), small (0.2–0.5), medium (0.5–0.8), and large (>0.8) (Cohen, 2013).

Based on the aforementioned procedures, two separate three-level random-effects models were constructed to compare the effects of LV-HIIT versus no additional-exercise control groups and LV-HIIT versus MICT. Parameter estimation was conducted using restricted maximum likelihood (REML), with cross-validation performed via maximum likelihood (ML) to ensure the robustness of results. In the primary analyses, because most studies reported multiple outcome measures, a three-level random-effects model was employed to account for the dependency among effect sizes within studies. In subsequent analyses focusing on single outcome indicators, each study contributed only one effect size, eliminating within-study correlations; thus, the model degenerated into a two-level random-effects structure during estimation. The statistical significance and 95% confidence intervals (CI) for individual coefficients were evaluated based on the t-distribution (Jukic et al., 2023). All analyses were performed in R using the “metafor” package (version 4.3.0; R Core Team, Vienna, Austria). Several statistical indicators can be used to assess heterogeneity (e.g., Cochrane’s Q, I^2^, tau^2^, and tau), but most methodological guidelines and textbooks recommend I^2^ as the primary index of heterogeneity (Nakagawa et al., 2017). Heterogeneity was assessed using the I^2^ statistic, which was interpreted as follows: <25% indicated unimportant heterogeneity, 25%–50% moderate heterogeneity, 50%–75% substantial heterogeneity, and >75% considerable heterogeneity (Cumpston et al., 2019). In the subsequent analyses, K was used to denote the number of included studies.

To explore potential sources of heterogeneity and moderators, subgroup analyses and meta-regression analyses were conducted respectively for categorical and continuous variables (Hopkins and Batterham, 2018). The subgroup analyses were performed from two perspectives: population characteristics and intervention characteristics. Population characteristics included age group (≤12 years and >12 years) and weight status (normal and overweight/obese). Intervention characteristics included intervention duration (≤8 weeks and >8 weeks) and total high-intensity exercise time (≤5 min and >5 min) (Yin et al., 2024e). In addition, continuous variables such as the number of intervention weeks, total training sessions, recovery interval per session (min), number of repetitions, duration of each high-intensity bout (min), and duration per repetition (s) were further examined through linear meta-regression analyses. All regression models were fitted within a three-level random-effects framework to account for the dependency among effect sizes within studies. Statistical significance was set at p < 0.05.

### 2.6 Publication bias and sensitivity analysis

To examine potential publication bias among the included studies, funnel plots were generated (Peters et al., 2008), and Egger’s regression asymmetry test was performed for quantitative assessment (Egger et al., 1997). Generally, these tests were conducted only when at least 10 studies were included, in order to ensure the robustness of the results (Sterne et al., 2011). The funnel plot visually displays the distribution of effect sizes against their standard errors to assess the symmetry of the results, providing an initial qualitative indication of publication bias. Egger’s regression test statistically evaluates the asymmetry of the funnel plot; if p > 0.05, no significant publication bias is considered present. By combining these two methods, publication bias can be more comprehensively assessed from both subjective and objective perspectives.

In addition to funnel plots and Egger’s test, a trim-and-fill method is often used to adjust for potential publication bias by imputing hypothetical missing studies to achieve funnel plot symmetry. However, this method remains methodologically controversial, particularly when the number of included studies is small or heterogeneity among studies is high, as it may lead to unstable or overcorrected results. Therefore, the trim-and-fill method was not further applied in this study. This limitation will be acknowledged in the Discussion section, and caution will be exercised when interpreting and generalizing the results.

Multiple sensitivity analyses were conducted in this study. First, Cook’s distance (Viechtbauer and Cheung, 2010) and studentized residuals (Atkinson et al., 1983) were used to identify potential outliers or studies exerting disproportionate influence on the overall effect size. Studies exhibiting excessively high influence or abnormal residuals within the model were flagged as potential outliers. Second, a leave-one-study-out (LOSO) approach (Meng et al., 2024) was applied to further assess the robustness of the findings, whereby each study was sequentially removed, and the pooled effect size was recalculated to observe any substantial changes in the overall estimate. If the overall effect remained consistent after excluding any individual study, the results were considered robust.

## 3 Results

### 3.1 Search results

**FIGURE 1:**
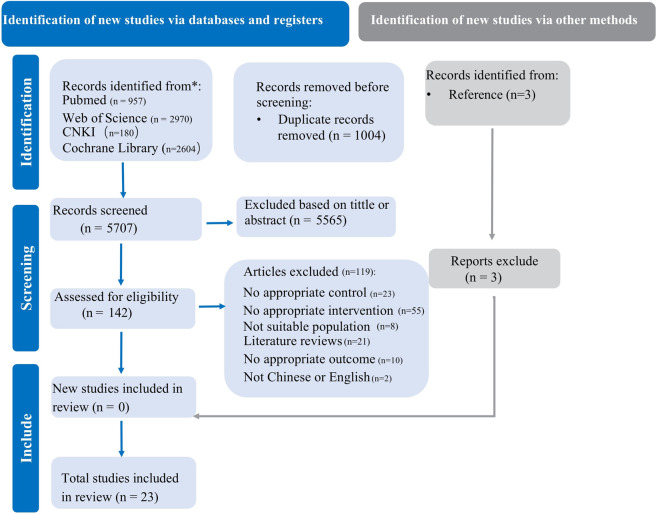
PRISMA flow diagram for included and excluded study.

A systematic search of four databases was conducted, yielding a total of 5,707 records after duplicate removal. Subsequently, 142 full-text articles were screened for eligibility, and 23 studies (Buchan et al., 2013; Lau et al., 2015; Cvetkovic et al., 2018; Liang and Hao, 2018; Alizadeh and Safarzade, 2019a; Alizadeh and Safarzade, 2019b; Alonso-Fernandez et al., 2019; Martin-Smith et al., 2019; Miguet et al., 2020; Yuan, 2021; Cao et al., 2022a; Julian et al., 2022; Leite et al., 2022; Cao et al., 2022b; Cao et al., 2023; Cao et al., 2024; Abassi et al., 2023; Li et al., 2023; González-Gálvez et al., 2024; Jovanovic et al., 2024; Ketelhut et al., 2024; Su et al., 2024; Su et al., 2025) were finally included in this systematic review and meta-analysis (Figure 1).

### 3.2 Study characteristics

A total of 996 participants were included across all studies, of whom 246 were female. 6 studies involved participants with normal weight, and 17 studies included those who were overweight or obese.

Four studies compared the effects of LV-HIIT with MICT alone (Liang and Hao, 2018; Miguet et al., 2020; Su et al., 2024; Su et al., 2025), and five studies compared LV-HIIT, MICT, and control groups (Cao et al., 2022a; Julian et al., 2022; Leite et al., 2022; Cao et al., 2022b; Li et al., 2023). Nine studies examined the effects of LV-HIIT compared with control groups only (Buchan et al., 2013; Alizadeh and Safarzade, 2019a; Alizadeh and Safarzade, 2019b; Alonso-Fernandez et al., 2019; Martin-Smith et al., 2019; Yuan, 2021; Cao et al., 2023; Jovanovic et al., 2024; Ketelhut et al., 2024). In addition, two studies compared LV-HIIT with MIIT (Abassi et al., 2023; Cao et al., 2024), while two other studies involved comparisons with different exercise modalities—one compared LV-HIIT with SSG (Cvetkovic et al., 2018), and another compared LV-HIIT with SIT (González-Gálvez et al., 2024). Notably, running was the most commonly used exercise modality, followed by cycling, Tabata-style bodyweight training, and finally, 6 min game-based interval protocols.

The intervention duration ranged from 4 to 16 weeks, with 12 weeks being the most common duration (K = 12). Training frequency varied from 2 to 4 sessions per week, with 3 sessions per week being the most frequently adopted (K = 17). Detailed characteristics of the included studies are presented in Table 2.

**TABLE 2:** The baseline characteristics of included studies.

Study	Population	Age (year)	Group	Group(n)	Protocol	Duration (weeks)	Frequency (days/week)
Su et al. (2024)	Obese adolescents; n = 44 (0 female)	15 ± 1	LV-HIIT	22	10 × 1-min running at 85%-95%HRpeak, interspersed with 10 × 2-min at (60%-70%HRpeak),with an RPE of 16–17	8	3
		14 ± 1	MICT	22	35-min running at 65%-75%HRpeak		
Julian et al. (2022)	Obese adolescents; n = 49 (29 female)	13.0 ± 1.1	LV-HIIT	19	15 × 30s cycling at 85%-95%HRpeak+30s active recovery free but compulsory pedaling + RT exercises	16	4
		13.0 ± 0.8	MICT	19	45-min running at 60%HRpeak + RT exercises		
		13.2 ± 1.0	CON	11	No extra exercise		
Su et al. (2024)	Normal adolescents; n = 24 (0 female)	12.83 ± 0.83	LV-HIIT	12	A 6-week intervention of 3 sessions per week, consisting of 4–6 × 30 s all-out cycling bouts at 7.5% body mass resistance with 4 min recovery	6	3
		13.33 ± 0.89	MICT	12	30–60 min cycling at 65%VO_2_peak		
Miguet et al. (2020)	Obese adolescents; n = 43 (31 female)	13.6 ± 1.5	LV-HIIT	22	15 × 30 s cycling at 85%-95%HRpeak+30s active recovery free but compulsory pedaling	16	4
		13.6 ± 1.5	MICT	21	45-min running at 60%HRpeak		
Leite et al. (2022)	Obese children; n = 56 (0 female)	12.84 ± 1.87	LV-HIIT	20	2 sets×8 × 30 s running/cycling at 100%MAS Recovery:60s; rest between sets:4min	12	3
		12.79 ± 1.56	MICT	20	45 min indoor cycling 35–55%HRR 45 min outdoor walking/running 35–55%HRR		
		12.58 ± 1.76	CON	16	No extra exercise		
Martin-Smith et al. (2019)	Normal adolescents; n = 52 (20 female)	17 ± 0.3	LV-HIIT	22	1 set×5–6 × 30 s all-out 20 m shuttle runs interspersed with 30 s passive walk	4	3
		16.80 ± 0.5	CON	30	No extra exercise		
Cao et al. (2022a)	Overweight children; n = 60 (30 female)	11.2 ± 0.9	LV-HIIT	20	3 sets×8 × 15s running at 100%–120% MAS, interspersed with 15s running at 50%MAS; rest between sets:3 min	12	3
		10.9 ± 0.8	MICT	20	20–40 min running at 60–70%MAS Every 4 weeks, the duration increases by 10 min and the intensity increases by 10%		
		10.9 ± 0.9	CON	20	No extra exercise		
Cao et al. (2023)	Obese children; n = 25 (n/a female)	11.0 ± 0.4	LV-HIIT	13	2 sets×8 × 15 s at 100% MAS, interspersed with 15 s at 50% MAS; 2 min rest between sets	12	3
		11.0 ± 0.4	CON	12	No extra exercise		
Cao et al. (2024)	Overweight children; n = 42 (0 female)	12.4 ± 0.4	LV-HIIT	14	3 sets×8 × 15s running at 100% MAS, interspersed with 15s recovery; rest between sets: 3 min	12	3
		12.1 ± 0.6	MIIT	14	3 sets×8 × 15s running at 80% MAS, interspersed with 15s recovery; rest between sets: 3 min		
		12.4 ± 0.5	CON	14	No extra exercise		
Li et al. (2023)	Overweight children; n = 60 (30 female)	11.0 ± 0.8	LV-HIIT	20	3 sets×8 × 15s running at 100%–120% MAS, interspersed with 15s running at 50%MAS; rest between sets:3 min	12	3
		11.0 ± 0.8	MICT	20	20–40 min running at 60–70%MAS; Every 4 weeks, the duration increases by 10 min and the intensity increases by 10%		
		11.0 ± 0.8	CON	20	No extra exercise		
Liang and Hao (2018)	Obese children; n = 56 (0 female)	n/a	LV-HIIT	9	60 s running at 100%speed, interspersed with 3 min running at 50%speed	12	3
		n/a	MICT	9	30–60 min running at 80%HRpeak Every 3 weeks, the duration increases by 10 min		
Abassi et al. (2023)	Obese adolescents; n = 38 (38 female)	16.4 ± 1.2	LV-HIIT	13	2 sets×6–8 × 30s running at 100%–110% MAS, interspersed with 30s at 50% MAS; rest between sets: 4min	12	3
		16.4 ± 1.2	MIIT	13	2 sets×6–8 × 30 s running at 60%–80% MAS, interspersed with 30 s at 50% MAS; rest between sets: 4 min		
		16.4 ± 1.2	CON	12	No extra exercise		
Yuan (2021)	Overweight adolescents; n = 40 (0 female)	16.1 ± 1.2	LV-HIIT	20	1–3 weeks:2sets×5 × 30 s cycling at 100%MAP interspersed with 30 s cycling at 50%MAP 4–6 weeks:3sets×6 × 30 s cycling at 100%MAP interspersed with 30 s cycling at 50%MAP 7–9 weeks:4sets×7 × 30 s cycling at 100%MAP interspersed with 30 s cycling at 50%MAP 10–12 weeks:5sets×8 × 30 s cycling at 100%MAP interspersed with 30 s cycling at 50%MAP	12	3
		15.±1.2	CON	20	No extra exercise		
Cvetkovic et al. (2018)	Overweight children; n = 35 (0 female)	11–13	LV-HIIT	11	1–4 weeks:3sets×5 × 10 s running at 100%MAS interspersed with 10 s recovery 5–8 weeks:3sets×8 × 15s running at 100%MAS interspersed with 15s recovery 9–12 weeks:3sets×10 × 20 s running at 100%MAS interspersed with 20 s recovery	12	3
		11–13	FOOTBALL	10	A relative pitch area of 80 m^2^ per player and length to width aspect ratio of 2:1 Football game 4 × 8 min playing interspersed with 2 min recovery		
		11–13	CON	14	No extra exercise		
Buchan et al. (2013)	Normal adolescents; n = 89 (25 female)	16.7.±0.6	LV-HIIT	42	1 × 4–6 × 30-s all-out 20-m shuttle runs, interspersed with 30-s recovery 20-s in week 7	7	3
		16.7.±0.6	CON	47	No extra exercise		
Lau et al. (2015)	Overweight children; n = 48 (12 female)	10.4 ± 0.9	LV-HIIT	15	12 × 15s running at 120% MAS, interspersed with 15s recovery	6	3
		10.4 ± 0.9	LIIT	21	16 × 15s running at 100% MAS, interspersed with 15s recovery		
		10.4 ± 0.9	CON	12	No extra exercise		
Alizadeh and Safarzade (2019a)	Overweight adolescents; n = 20 (0 female)	16.2 ± 1.3	LV-HIIT	10	4–6 × 30s running at 90% HRmax, interspersed with 30s recovery	6	3
		16.2 ± 1.3	CON	10	No extra exercise		
Alizadeh and Safarzade (2019b)	Overweight adolescents; n = 20 (0 female)	18.0 ± 1.5	LV-HIIT	10	4–6 × 30s running at 90% HRmax, interspersed with 30s recovery	6	3
		18.0 ± 1.5	CON	10	No extra exercise		
Jovanovic et al. (2024)	Normal adolescents; n = 60 (0 female)	16.33 ± 0.62	LV-HIIT	30	2 sets×8 × 20 s Tabata body-weight/running drills at 80%–90% HRmax, interspersed with 10 s rest; rest between sets: 1 min	12	2
		16.33 ± 0.62	CON	30	No extra exercise		
Cao et al. (2022b)	Obese children; n = 36 (0 female)	11.4 ± 0.8	LV-HIIT	12	2 sets×8 × 15 s running at 90%–100% MAS, interspersed with 15 s at 50% MAS	12	3
		11.2 ± 0.7	MICT	11	30 min continuous running at 60%–70% MAS, weeks 1–4: 60%, 5–8: 65%, 9–12: 70%)		
		11.0 ± 0.7	CON	13	No extra exercise		
Ketelhut et al. (2024)	Normal children; n = 40 (17 female)	11.0 ± 0.6	LV-HIIT	20	2 × 6-min game-based HIIT blocks 20 s-2 min bouts with 30–90 s active/passive rest; 3-min break between blocks	12	2
		11.0 ± 0.7	CON	20	No extra exercise		
González-Gálvez et al. (2024)	Overweight adolescents; n = 32 (14 female)	12.51 ± 0.75	SIT	9	6 sets×1 × 60 s running at 90%–95% HRR, interspersed with 60 s at 50%–55% HRR	8	2
		12.51 ± 0.75	LV-HIIT	11	3 sets×1 × 120 s running at 80%–85% HRR, interspersed with 120 s at 50%–55% HRR		
		12.51 ± 0.75	CON	12	No extra exercise		
Alonso-Fernandez et al. (2019)	Normal adolescents; n = 26 (n/a female)	15–16	LV-HIIT	13	Tabata-style HIIT progressively from 1 × 8 × 20 s all-out/10 s rest in week 1, to 2 × 8 × 20 s/10 s rest with 1-min between sets in weeks 6–7	9	2
		15–16	CON	13	No extra exercise		

### 3.3 Effects of LV-HIIT vs. CON

First, a three-level meta-analysis was conducted for all included outcome measures. The overall main effect showed that, compared with the no additional-exercise control group, LV-HIIT significantly improved overall body composition and cardiovascular health in children and adolescents (SMD = −0.39, 95% CI [−0.66, −0.12], p < 0.01). High within-study heterogeneity was observed (I^2^ level 2 = 85%, I^2^ level 3 = 0%, PI [-2.35, 1.57]), indicating the necessity of further analyses by individual outcome indicators to explore their potential influence on the overall effect. The forest plot of the three-level main effect is presented in Supplementary Datasheet S2.

Regarding body composition outcomes, the meta-analysis showed that, compared with the no additional-exercise control group, LV-HIIT had a significant effect on BMI, with moderate heterogeneity among studies (K = 17, g = −1.24, 95% CI [-2.03, 0.68], I^2^ = 44.7%, PI [-4.45, 1.97], p < 0.01, moderate GRADE). A significant effect was also observed for fat mass, with moderate heterogeneity (K = 8, g = −0.99, 95% CI [-1.71, - 0.28], I^2^ = 38.4%, PI [-2.91, 0.92], p = 0.01, low GRADE). LV-HIIT significantly reduced body fat, with very low heterogeneity across studies (K = 11, g = −0.89, 95% CI [-1.16, −0.62], I^2^ = 0%, PI [-1.16, −0.62], p < 0.01, low GRADE). A significant reduction was also found in waistline, with very low heterogeneity (K = 7, g = −0.42, 95% CI [-0.73, −0.11], I^2^ = 2.6%, PI [-0.79, −0.05], p < 0.01, low GRADE). Similarly, LV-HIIT produced a moderate effect on bodyweight, with very low heterogeneity (K = 13, g = −0.34, 95% CI [-0.57, −0.12], I^2^ = 6%, PI [-0.72, 0.03], p < 0.01, moderate GRADE). However, no significant effect was observed for fat-free mass, with very low heterogeneity (K = 8, g = −0.03, 95% CI [-0.34, 0.29], I^2^ = 0%, PI [-0.34, 0.29], p = 0.83, low GRADE).

**FIGURE 2:**
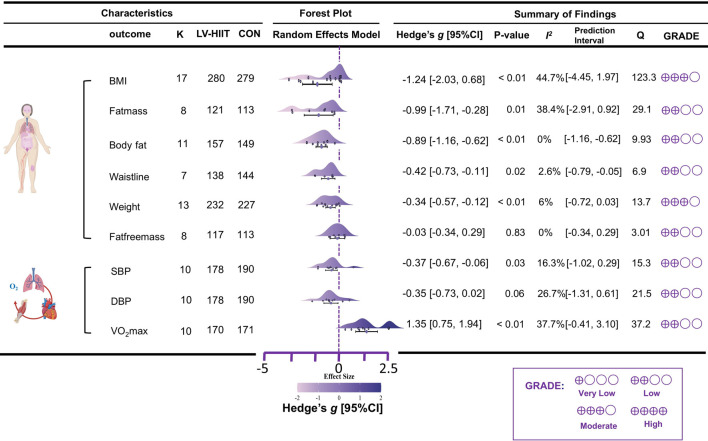
Primary pooled effect sizes for LV-HIIT vs. CON.

Regarding cardiovascular health outcomes, the meta-analysis indicated that, compared with the no additional-exercise control group, LV-HIIT had a significant effect on SBP, with low heterogeneity across studies (K = 10, g = −0.37, 95% CI [-0.67, −0.06], I^2^ = 16.3%, PI [−1.02, 0.29], p = 0.03, low GRADE). However, no significant effect was observed for DBP, with similarly low heterogeneity (K = 10, g = −0.35, 95% CI [-0.73, 0.02], I^2^ = 26.7%, PI [−1.31, 0.61], p = 0.06, low GRADE). In contrast, LV-HIIT produced a significant improvement in VO_2_max, with moderate heterogeneity among studies (K = 10, g = 1.35, 95% CI [0.75, 1.94], I^2^ = 37.7%, PI [−0.41, 3.10], p < 0.01, low GRADE). All corresponding details are presented in Figure 2.

Secondly, subgroup analyses were conducted to examine whether age, weight status, intervention duration, and high-intensity training bout duration influenced the effects of LV-HIIT. The results showed that, compared with participants with normal BMI, LV-HIIT had a greater effect on reducing bodyweight in those who were overweight or obese (g = −0.50, p < 0.01), with a significant difference between subgroups (p = 0.039). For SBP, LV-HIIT produced a more pronounced improvement among children (g = −0.65, p < 0.01), and the subgroup difference was significant (p = 0.024). In addition, interventions lasting longer than 8 weeks demonstrated greater SBP improvement (g = −0.59, p < 0.01), with a significant subgroup difference (p = 0.027). Regarding DBP, longer intervention durations (>8 weeks) were correlated with a more significant reduction (g = −0.61, p < 0.01), and subgroup differences were significant (p = 0.014). For VO_2_max, LV-HIIT showed a greater improvement among overweight or obese participants (g = 1.68, p < 0.01), with a significant subgroup difference (p = 0.047). All detailed results are presented in Table 3.

**TABLE 3:** Subgroup analysis of Effects of LV-HIIT vs. CON.

Outcomes	Subgroup	K	Hedges’ *g*	95% CI	*p*_v_	*I* ^2^	*p*_b_
BMI	≤12 years	6	−1.38	[-2.77, 0]	0.05	81.8%	0.793
	>12 years	11	−1.17	[-2.20, −0.13]	0.03	92.2%	
	Normal	3	0.01	[-1.73, 1.74]	0.99	0%	0.114
	OW/OB	14	−1.51	[-2.35, −0.67]	0.002	89.1%	
	≤8 weeks	5	−1.85	[-3.42, −0.27]	0.02	94.7%	0.354
	>8 weeks	12	−1.02	[-1.99, −0.05]	0.04	81.2%	
	≤5 min	6	−2.22	[-3.53, −0.90]	0.003	92.8%	0.068
	>5 min	11	−0.74	[-1.66, 0.19]	0.11	77.9%	
Fatmass	≤12 years	5	−1.36	[-2.20, −0.53]	0.007	78.2%	0.127
	>12 years	3	−0.40	[-1.44, 0.63]	0.38	0%	
	Normal	1	−0.21	[-2.30, 1.89]	0.82	N/a	0.364
	OW/OB	7	−1.11	[-1.91, −0.31]	0.02	76.9%	
	≤5 min	2	−1.46	[-3.03, 0.11]	0.06	82.4%	0.440
	>5 min	6	−0.85	[-1.73, 0.02]	0.05	77.5%	
Bodyfat	≤12 years	4	−0.91	[-1.45, 1.06]	0.76	86%	0.720
	>12 years	7	−0.88	[-0.97, 0.09]	0.10	56%	
	Normal	1	−0.26	[-1.15, 0.63]	0.52	N/A	0.127
	OW/OB	10	−0.96	[-1.25, −0.67]	<0.01	0%	
	≤8 weeks	2	−1.60	[-2.42, −0.78]	<0.01	0%	0.068
	>8 weeks	9	−0.80	[-1.09, −0.51]	<0.01	0%	
	≤5 min	4	−1.24	[-1.76, −0.71]	<0.01	0%	0.114
	>5 min	7	−0.76	[-1.08, −0.44]	<0.01	0%	
Waistline	≤12 years	3	−0.72	[-1.28, −0.16]	0.02	39.4%	0.149
	>12 years	4	−0.28	[-0.65, 0.10]	0.12	0%	
	Normal	2	−0.23	[-0.68, 0.23]	0.25	0%	0.208
	OW/OB	5	−0.58	[-1.01, −0.15]	0.02	18.3%	
	≤8 weeks	2	−0.23	[-0.68, 0.23]	0.25	0%	0.208
	>8 weeks	5	−0.58	[-1.01, −0.15]	0.02	18.3%	
	≤5 min	3	−0.36	[-0.84, 0.11]	0.11	0%	0.625
	>5 min	4	−0.50	[-1.00, −0.01]	0.05	16.8%	
Weight	≤12 years	4	−0.43	[-0.89, 0.03]	0.06	0%	0.626
	>12 years	9	−0.31	[-0.58, −0.05]	0.03	20.1%	
	Normal	3	−0.04	[-0.38, 0.29]	0.78	0%	0.039
	OW/OB	10	−0.50	[-0.77, −0.24]	<0.01	0%	
	≤8 weeks	3	−0.21	[-0.65, 0.23]	0.32	26.4%	0.451
	>8 weeks	10	−0.39	[-0.66, −0.12]	<0.01	10.1%	
	≤5 min	4	−0.32	[-0.74, 0.10]	0.13	34.8%	0.846
	>5 min	9	−0.36	[-0.65, −0.07]	0.02	10%	
Fatfreemass	≤12 years	4	−0.06	[-0.51, 0.40]	0.77	0%	0.840
	>12 years	4	0.00	[-0.47, 0.47]	0.99	0%	
	Normal	2	−0.02	[-0.74, 0.70]	0.95	16.8%	0.965
	OW/OB	6	−0.03	[-0.40, 0.33]	0.83	0%	
	≤8 weeks	1	−0.38	[-1.47, 0.71]	0.42	N/A	0.437
	>8 weeks	7	0.01	[-0.34, 0.35]	0.97	0%	
	≤5 min	1	0.03	[-0.95, 1.01]	0.95	N/A	0.884
	>5 min	7	−0.04	[-0.38, 0.31]	0.80	0%	
SBP	≤12 years	6	−0.65	[-1.00, −0.30]	<0.01	0%	0.024
	>12 years	4	−0.06	[-0.40, 0.28]	0.71	0%	
	Normal	3	−0.20	[-0.69, 0.28]	0.36	0%	0.352
	OW/OB	7	−0.47	[-0.87, 0.07]	0.03	52.5%	
	≤8 weeks	3	0.00	[-0.38, 0.38]	0.98	27.9%	0.027
	>8 weeks	7	−0.59	[-0.91, −0.27]	<0.01	0%	
	≤5 min	4	−0.34	[-0.86, 0.17]	0.16	13.1%	0.891
	>5 min	6	−0.39	[-0.84, 0.07]	0.09	52.1%	
DBP	≤12 years	6	−0.49	[-0.99, 0.02]	0.06	0%	0.387
	>12 years	4	−0.18	[-0.77, 0.41]	0.51	82.6%	
	Normal	3	−0.11	[-0.72, 0.49]	0.68	49.9%	0.289
	OW/OB	7	−0.49	[-0.95, −0.03]	0.04	52.1%	
	≤8 weeks	3	0.15	[-0.29, 0.58]	0.46	35.1%	0.014
	>8 weeks	7	−0.61	[-0.95, −0.26]	<0.01	0%	
	≤5 min	4	−0.11	[-0.64, 0.42]	0.65	27%	0.207
	>5 min	6	−0.53	[-1.01, −0.06]	0.03	58.3%	
VO2max	≤12 years	3	2.08	[1.08, 3.08]	<0.01	62%	0.076
	>12 years	7	1.05	[0.32, 1.71]	<0.01	68.5%	
	Normal	3	0.62	[-0.22, 1.47]	0.13	49%	0.047
	OW/OB	7	1.68	[1.07, 2.29]	<0.01	65.8%	
	≤8 weeks	2	1.02	[-0.40, 2.44]	0.14	0%	0.568
	>8 weeks	8	1.43	[0.72, 2.15]	<0.01	80.1%	
	≤5 min	3	1.58	[0.41, 2.76]	0.02	61.9%	0.602
	>5 min	7	1.25	[0.50, 2.01]	<0.01	80.4%	

K: the total number of effects included in the pooled effect size; Hedges’ g: the effect size indicators used in the pooled; p_v_: overall pooled effect; p_b_: between subgroups differences; 95%CI: 95% confidence interval; I^2^: quantitative indicators of heterogeneity; BMI: body mass index; VO_2_max: Maximal Oxygen Uptake; DBP: diastolic blood pressure; SBP: systolic blood pressure; OW/OB, Overweight/Obese.

**FIGURE 3:**
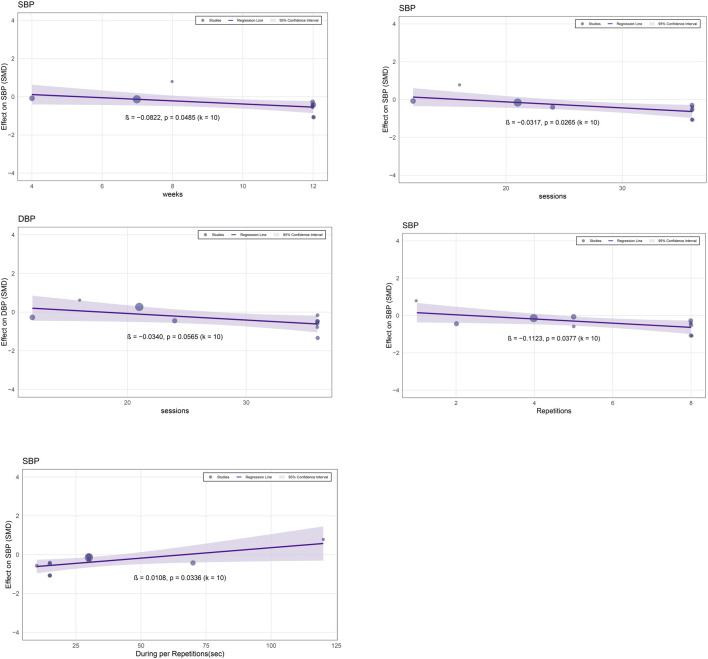
Meta-regression results of LV-HIIT vs. CON.

In addition, this study further examined the potential moderating effects of six continuous variables, including intervention weeks, total training sessions, recovery interval within each session, The number of repetitions in high-intensity interval training, duration of each high-intensity training bout, and duration per repetition. The meta-regression results showed a significant negative association between intervention weeks and improvements in SBP (β = −0.0822, p = 0.0485), indicating that a longer intervention duration enhanced the hypotensive effect of LV-HIIT. Total training sessions were also significantly negatively correlated with SBP improvement (β = −0.0317, p = 0.0265), suggesting that a greater number of sessions led to a more pronounced reduction in SBP. Although the association between total training sessions and DBP improvement showed a negative trend, it did not reach statistical significance (β = −0.0340, p = 0.0565), implying that increasing the number of sessions may contribute to DBP reduction, but the evidence remains insufficient. The number of repetitions in high-intensity interval training was significantly negatively correlated with SBP improvement (β = −0.1123, p = 0.0377), indicating that more repetitions were correlated with greater reductions in SBP. Conversely, the duration per repetition was significantly positively correlated with SBP improvement (β = 0.0108, p = 0.0336), suggesting that shorter repetition durations might be more effective for reducing SBP, whereas prolonged durations could weaken the hypotensive effect. Further details are presented in Figure 3 and Supplementary Datasheet S3.

### 3.4 Effects of LV-HIIT vs. MICT

A three-level meta-analysis was also conducted for all included outcome measures. The main effect revealed that, compared with MICT, LV-HIIT did not produce a significant improvement in overall body composition and cardiovascular health among children and adolescents (SMD = −0.03, 95% CI [−0.29, 0.24], p = 0.845). Moderate within-study heterogeneity was observed (I^2^ level 2 = 49.1%, I^2^ level 3 = 0%, PI [-0.81, 0.76]), indicating the necessity of further analyses by individual outcome indicators to explore their potential influence on the overall effect. The forest plot of the three-level main effect is presented in Supplementary Datasheet S4.

Regarding body composition outcomes, the meta-analysis showed that, compared with MICT, LV-HIIT had no significant effect on BMI, with very low heterogeneity among studies ((K = 9, g = −0.14, 95% CI [-0.41, 0.13], I^2^ = 0%, PI [-0.41, 0.13], p = 0.27, moderate GRADE). No significant effect was observed for fat mass, with very low heterogeneity (K = 7, g = 0.12, 95% CI [-0.22, 0.46], I^2^ = 0%, PI [-0.22, 0.46], p = 0.42, low GRADE). Similarly, LV-HIIT did not significantly affect body fat, with low heterogeneity (K = 5, g = 0.03, 95% CI [−0.51, 0.57], I^2^ = 11.4%, PI [−0.78, 0.83], p = 0.89, very low GRADE). No significant difference was found in waistline, with moderate heterogeneity across studies (K = 5, g = −0.28, 95% CI [−1.03, 0.47], I^2^ = 28.8%, PI [−1.77, 1.21], p = 0.36, very low GRADE). Similarly, LV-HIIT showed no significant effect on bodyweight, with low heterogeneity (K = 7, g = −0.06, 95% CI [-0.38, 0.25], I^2^ = 11.4%, PI [−0.78, 0.83], p = 0.89, low GRADE). Furthermore, no significant effect was observed for fat-free mass, with very low heterogeneity (K = 6, g = 0.14, 95% CI [−0.22, 0.50], I^2^ = 0%, PI [−0.22, 0.50], p = 0.35, very low GRADE).

**FIGURE 4:**
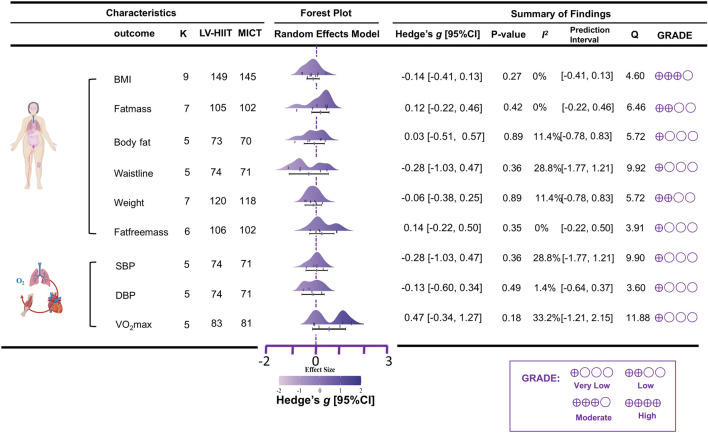
Primary pooled effect sizes for LV-HIIT vs. MICT.

Regarding cardiovascular health outcomes, the meta-analysis showed that, compared with MICT, LV-HIIT had no significant effect on SBP, with moderate heterogeneity among studies (K = 5, g = −0.28, 95% CI [−1.03, 0.47], I^2^ = 28.8%, PI [−1.77, 1.21], p = 0.36, very low GRADE). No significant effect was observed for DBP, with very low heterogeneity (K = 5, g = −0.13, 95% CI [−0.60, 0.34], I^2^ = 1.4%, PI [−0.64, 0.37], p = 0.49, very low GRADE). Similarly, LV-HIIT did not significantly affect VO_2_max, with moderate heterogeneity across studies (K = 5, g = 0.47, 95% CI [-0.34, 1.27], I^2^ = 33.2%, PI [−1.21, 2.15], p = 0.18, very low GRADE). All corresponding details are presented in Figure 4.

Secondly, the subgroup analysis results indicated that age, weight status, intervention duration, and high-intensity training bout duration were not significant moderators of the effects between LV-HIIT and MICT (p > 0.05). All corresponding details are presented in Supplementary Datasheet S5. In addition, due to the limited number of eligible studies, further meta-regression analyses could not be performed.

### 3.5 Descriptive comparison of LV-HIIT with other exercise interventions

In addition to comparisons with control groups and MICT, several studies also examined the effects of LV-HIIT relative to other exercise modalities. Due to the limited number of relevant studies, only a descriptive summary is provided. Two studies compared LV-HIIT with MIIT: one among overweight children and another among obese adolescents. Both adopted running-based protocols consisting of 15–30 s sprints with a 1:1 work-to-rest ratio, where high-intensity bouts were performed at 100%–110% Maximal Aerobic Speed (MAS) and moderate-intensity bouts at 60%–80% MAS. Regarding body composition, both studies reported significant differences, indicating that LV-HIIT yielded greater improvements than MIIT, and LV-HIIT also demonstrated superior enhancement in VO_2_max. Another study investigated the effects of LV-HIIT and SSG in overweight children. The LV-HIIT group performed 10–20 s running sprints at 100% MAS with a 1:1 work-to-rest ratio, while the SSG group engaged in soccer-based games within an 80 m^2^ pitch (length-to-width ratio 2:1) for 4 × 8 min with 2 min recovery intervals. Both LV-HIIT and SSG tended to improve bodyweight and BMI, but intergroup differences were not significant. Similarly, both showed improvements in SBP and DBP without significant differences. Another study compared low-volume SIT (120% MAS) with a relatively lower-intensity HIIT protocol (100% MAS) among overweight children, showing that both modalities benefited bodyweight but without significant group differences. Additionally, one study compared high-volume SIT (HV-SIT) and LV-HIIT among overweight adolescents, finding that LV-HIIT significantly improved fat mass, DBP, and VO_2_max but not BMI, whereas HV-SIT produced significant improvements only in fat mass.

### 3.6 Risk of bias and certainty of evidence

**FIGURE 5:**
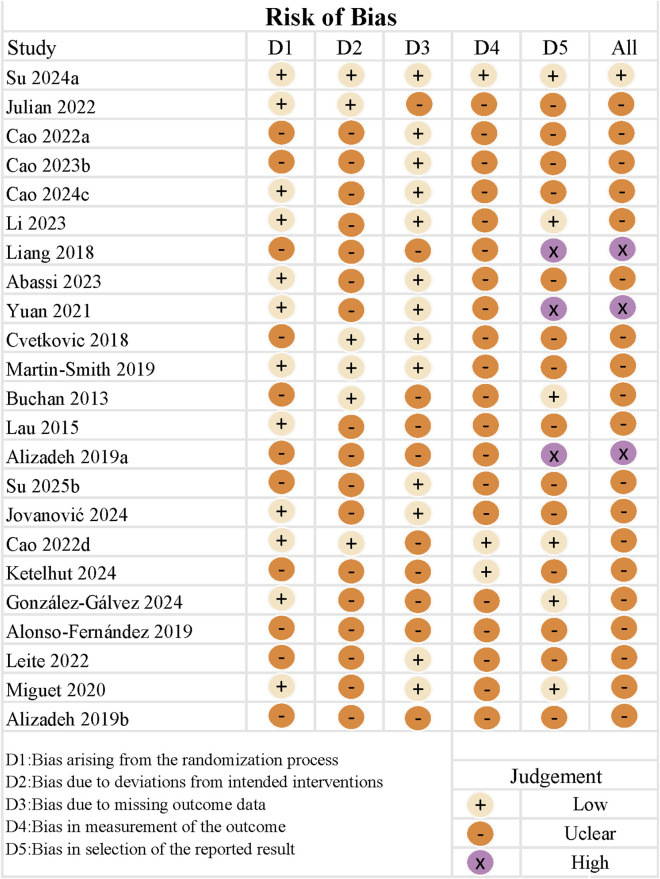
Risk of bias for the included studies.

As shown in Figure 5, most studies exhibited an overall moderate risk of bias, with three studies rated as having a high overall risk (Liang and Hao, 2018; Alizadeh and Safarzade, 2019a; Yuan, 2021) and one study rated as low risk (Su et al., 2024). Among the five assessed domains, the highest proportion of low-risk judgments (56%) was observed for bias due to missing outcome data, while the most frequent source of moderate risk was bias in outcome measurement. High-risk judgments were primarily concentrated in the domain of selective reporting bias (13%). These findings suggest that most studies adequately controlled for attrition and missing data; however, blinding during outcome assessment was often lacking, and a small number of studies may have selectively reported favorable results or omitted non-significant findings.

To comprehensively evaluate the certainty of evidence for each primary outcome, the GRADE approach was applied to rate the overall quality of evidence (see Supplementary Datasheet S6). In the comparison between LV-HIIT and no additional-exercise controls, 78% of the outcomes were rated as low-quality evidence, primarily due to the risk of bias in the included studies. In addition, several indicators (e.g., waistline, fat mass, fat-free mass) were affected by small sample sizes, leading to imprecision and a potential risk of small-study bias. In the comparison between LV-HIIT and MICT, several indicators (e.g., waistline, VO_2_max, SBP, DBP, body fat, and fat-free mass) were rated as very low-quality evidence, largely due to small sample bias and methodological limitations in study design. Therefore, these GRADE ratings highlight the need for future high-quality research to strengthen the evidence base in this field.

### 3.7 Publication bias and sensitivity analysis

Publication bias was assessed using funnel plots in combination with Egger’s regression test to examine the potential risk of bias among the included studies regarding health outcomes. It should be noted that the power of funnel plots and Egger’s test is limited when fewer than ten studies are included; therefore, only outcomes with ≥10 studies were subjected to statistical testing. As shown in Supplementary Datasheet S7, Egger’s tests indicated statistically significant results for VO_2_max (p = 0.026), BMI (p < 0.01), weight (p = 0.05), body fat (p = 0.045), and fat mass (p = 0.043), suggesting possible small-study effects or publication bias. In the comparison between LV-HIIT and MICT, funnel plots and Egger’s regression tests were performed for each health-related outcome (Supplementary Datasheet S8). However, since the number of included studies for each outcome was fewer than ten, the statistical power of these tests was limited, and the results should be interpreted descriptively. Visually, most funnel plots appeared relatively symmetrical, although some outcomes (e.g., VO_2_max and waistline) yielded borderline significant p-values (both 0.010) in Egger’s test, suggesting a potential small-study effect. Nevertheless, given that Egger’s test can produce false-positive or false-negative findings when the number of studies is small, these results cannot be considered robust evidence of publication bias. Overall, no formal assessment of publication bias was conducted for the LV-HIIT versus MICT comparison; instead, funnel plots were used for visual inspection, and we emphasize the need for more large-scale studies to validate the robustness of the existing findings.

To assess the robustness of the results, sensitivity analyses were performed using Cook’s distance and studentized residuals to identify potential outlier studies. In the LV-HIIT versus control model, most outcome indicators did not identify potential outliers, and neither the direction nor the statistical significance of the pooled effects changed substantially, suggesting high robustness of the findings. For BMI, two studies were identified as potential outliers; after their exclusion, the effect size slightly decreased (g = −0.78) but remained significant (Alizadeh and Safarzade, 2019a; Alizadeh and Safarzade, 2019b). For SBP, one potential outlier was identified; removing it slightly attenuated the effect size (g = −0.28), though the result remained significant (Cao et al., 2022a). For fat-free mass, one study was identified as an outlier, and its exclusion caused a negligible increase in the effect size (g = −0.09), which remained significant (Leite et al., 2022). For body fat, one potential outlier was found; after removal, the effect size slightly decreased (g = −0.96) but retained significance (Alonso-Fernandez et al., 2019). For DBP, two potential outliers were detected; their exclusion did not alter the effect direction but led to statistical significance (g = −0.35) (Buchan et al., 2013; Leite et al., 2022), suggesting that results for these indicators should be interpreted with caution (Supplementary Datasheet S9). In the LV-HIIT versus MICT model, only one study was identified as a potential outlier for BMI, SBP, and weight, but its exclusion did not change the overall conclusion (Leite et al., 2022). Moreover, no substantial changes were observed in other outcomes, further supporting the robustness of the findings (Supplementary Datasheet S10).

Subsequently, a leave-one-study-out (LOSO) sensitivity analysis was conducted to further evaluate the robustness of the pooled effects. In the LV-HIIT versus control comparison, the results indicated that after removing two studies, the significance of SBP became non-significant (Li et al., 2023; Ketelhut et al., 2024), whereas the exclusion of two studies in DBP led to statistical significance (Buchan et al., 2013; González-Gálvez et al., 2024). These findings suggest that the results for these two outcomes should be interpreted with caution (Supplementary Datasheet S10). In the LV-HIIT versus MICT comparison, the pooled effect sizes and their statistical significance remained essentially unchanged regardless of which study was excluded, indicating that the overall findings of the present meta-analysis were robust (Supplementary Datasheet S11).

## 4 Discussion

### 4.1 Summaries of the findings

Our main findings can be summarized as follows: (1) LV-HIIT significantly improved BMI, fat mass, body fat, waistline, weight, SBP, and VO_2_max, whereas no significant effects were observed for DBP and fat-free mass. (2) There were no significant differences between LV-HIIT and MICT across all included indicators of body composition and cardiovascular health. (3) Compared with no additional exercise, LV-HIIT produced greater improvements in bodyweight and VO_2_max among children and adolescents with overweight or obesity. Moreover, intervention durations longer than 8 weeks resulted in greater reductions in SBP and DBP, particularly in children. (4) Longer intervention periods, higher training frequencies, and greater numbers of repetitions were correlated with more pronounced reductions in SBP and DBP, whereas extending the duration of each high-intensity repetition appeared to attenuate these antihypertensive effects.

### 4.2 Effects of LV-HIIT compared with control and MICT

According to our the meta-analyses, LV-HIIT was found to effectively improve body composition and cardiovascular health in children and adolescents compared with the no-exercise control group. Notably, large effect sizes (g > 0.8) were observed for BMI, fat mass, body fat, and VO_2_max. To date, no systematic review has specifically examined the effects of LV-HIIT in children and adolescents based on a definition of ≤10 min of accumulated high-intensity exercise per session. However, previous systematic reviews and meta-analyses have investigated the overall effects of HIIT in this population (Costigan et al., 2015; García-Hermoso et al., 2016; Cao et al., 2019; Cao et al., 2021; Liu et al., 2020; Liu et al., 2024; Yin et al., 2020; Men et al., 2023; Deng and Wang, 2024; Wang et al., 2024; Zheng et al., 2025), and our findings regarding cardiovascular health are generally consistent with theirs. Nevertheless, several earlier meta-analyses reported that HIIT did not significantly improve waistline, BMI, or body fat (Costigan et al., 2015; Thivel et al., 2019; Men et al., 2023). A closer inspection of those studies revealed that their inconsistent results might stem from the limited number of included studies and the methodological error of pooling HIIT - MICT comparisons together with HIIT - control comparisons. In contrast, our analysis clearly differentiated between these comparisons, applied GRADE evaluation to each outcome, and demonstrated low to moderate heterogeneity for body composition outcomes, with sensitivity analyses confirming the robustness of the results. In our previously published work, we have already shown that HIIT effectively improves BMI, waistline, and body fat (Zheng et al., 2025); consistent with those findings, significant effects were again observed in the present study. These results may indirectly suggest that reducing the total training volume in HIIT does not necessarily diminish its benefits for bodyweight management. The underlying mechanisms may involve mitochondrial adaptation induced by HIIT, which enhances fat oxidation capacity and extends post-exercise energy expenditure through elevated excess post-exercise oxygen consumption (EPOC) (Henríquez-Olguín et al., 2019; Valéria et al., 2021). Similar physiological adaptations have also been observed in studies examining the effects of LV-HIIT on cardiovascular health (Yin et al., 2024e). Therefore, it is plausible that training intensity and interval structure, rather than total exercise volume, play a more crucial role in driving improvements in body composition. Nonetheless, the long-term effects and mechanisms correlated with different training volumes warrant further investigation.

No statistically significant differences were found between LV-HIIT and MICT in terms of body composition and cardiovascular health, with LV-HIIT showing a slightly greater improvement in VO_2_max (g = 0.47, p = 0.18). However, from a practical perspective, previous research has indicated that LV-HIIT requires approximately 40% less time and energy expenditure compared with MICT (Ryan et al., 2020).

In exploring the moderating factors, our findings revealed that LV-HIIT interventions lasting longer than 8 weeks produced greater improvements in bodyweight, blood pressure, and VO_2_max among children and adolescents with overweight or obesity. The superior weight-reducing effect of LV-HIIT in these populations, compared with their normal-weight counterparts, may be attributed to the presence of insulin resistance and lipid metabolism dysfunction commonly observed in overweight or obese individuals. Previous research has indicated that LV-HIIT enhances mitochondrial enzyme activity, thereby improving insulin resistance (Fabbri et al., 2016), and promotes glycogen depletion and subsequent resynthesis, which contributes to improved post-exercise insulin sensitivity (Ryan et al., 2020). These metabolic adaptations are more likely to manifest as measurable reductions in bodyweight among overweight or obese individuals, whereas normal-weight participants exhibit smaller changes due to their near-normal baseline conditions. Moreover, longer intervention durations were correlated with greater improvements in cardiopulmonary fitness, particularly reflected in reduced blood pressure and enhanced VO_2_max in overweight or obese youth. This may be explained by the increased shear stress induced by high-intensity interval exercise, which stimulates endothelial nitric oxide (NO) release, improves vascular dilation, and decreases peripheral resistance (Short et al., 2012). In contrast, the limited improvement observed in normal-weight participants may be due to their higher baseline VO_2_max, leaving less room for physiological adaptation (Enríquez-del-Castillo et al., 2022). Additionally, our analysis found no moderating factors explaining the differences between LV-HIIT and MICT in either body composition or cardiopulmonary outcomes, which is consistent with previous research (García-Hermoso et al., 2016; Zheng et al., 2025). These results suggest that in children and adolescents, LV-HIIT and MICT provide comparable overall benefits for body composition and cardiopulmonary health, and that the choice of training modality may not be the decisive factor. Considering that LV-HIIT offers superior time efficiency while MICT has well-established efficacy, both can serve as feasible intervention strategies, with the optimal choice depending on individual characteristics and practical feasibility.

In exploring the dose - response relationship, our study identified a significant association between LV-HIIT and blood pressure improvement. Specifically, a greater number of intervention weeks, more training sessions, and more repetitions were all effective variables contributing to reductions in blood pressure. Notably, this study is the first to reveal a significant inverse dose - response relationship between the duration of each high-intensity repetition and the reduction in SBP among children and adolescents (β = 0.0108), suggesting that excessively prolonged high-intensity bouts may attenuate the antihypertensive benefits of LV-HIIT. Considering that cardiovascular regulation in children and adolescents is not yet fully developed and that their arterial compliance is relatively low, such physiological immaturity may amplify unfavorable blood pressure responses under extended high-intensity exertion. Therefore, exercise prescriptions for this population should avoid overly long high-intensity durations. Moreover, we found no significant difference between LV-HIIT and MICT in improving SBP, indicating that LV-HIIT can provide comparable blood pressure benefits within a shorter timeframe. This supports the notion that LV-HIIT, as a more time-efficient training modality, may be particularly suitable for children and adolescents with limited time availability or low exercise adherence. Nevertheless, due to the limited number of included studies, these findings should be interpreted cautiously and verified in future large-sample and long-term trials. Unfortunately, the small number of eligible studies prevented us from further exploring the dose - response relationship between LV-HIIT and MICT. Future high-quality randomized controlled trials are warranted to clarify the differential dose configurations and physiological advantages of these two training modalities, thereby providing stronger evidence for individualized exercise prescription. In addition, it should be noted that much of the mechanistic understanding of HIIT-induced cardiovascular adaptations (e.g., improvements in autonomic regulation, enhancements in vascular function, and increases in shear-stress–mediated nitric oxide release) has been derived primarily from adult populations. Children and adolescents differ from adults in several developmental physiological characteristics, including the maturity of the autonomic nervous system, arterial elasticity, metabolic regulation, and hormonal profiles (Logan et al., 2014). These differences may influence how young individuals respond to high-intensity interval exercise, suggesting that although the direction of adaptation appears similar to adults, the underlying mechanisms in youth may not be entirely the same. Future studies incorporating youth-specific physiological measures are needed to clarify the developmental pathways through which LV-HIIT exerts its cardiovascular benefits.

### 4.3 Comparison of LV-HIIT with other exercise interventions

Overall, the populations included in studies comparing LV-HIIT with other exercise modalities (such as MIIT, SSG, and HV-SIT) were overweight or obese children and adolescents. When compared with MIIT, LV-HIIT produced more pronounced improvements in body composition and cardiovascular health, suggesting that training intensity may be an important moderating factor influencing intervention efficacy. This observation is consistent with previous research indicating that the effects of interval training on body composition and cardiorespiratory function largely depend on key parameters such as intensity, frequency, and duration (Ouerghi et al., 2017). Furthermore, our findings imply that training volume may not be the decisive factor for health improvements, as MIIT can also yield positive effects to some extent. Thus, future studies could explore the potential application of low-volume MIIT in overweight or obese youth, particularly given its potentially higher exercise adherence and practical feasibility (Abassi et al., 2023). From a physiological perspective, moderate-intensity exercise can also improve body composition and cardiovascular health; however, the optimization of interval structure remains a crucial determinant of efficacy. In studies comparing LV-HIIT and SSG, SSG showed greater improvements in body composition, likely due to its typically longer duration (>20 min) (Milanović et al., 2015; Cvetkovic et al., 2018), which results in higher energy expenditure. Considering that participants in these studies were overweight or obese, this may explain the superior outcomes observed with SSG. Nevertheless, LV-HIIT, characterized by higher time efficiency and flexibility, may be a more practical option for real-world implementation. Additionally, studies comparing SIT (120% MAS) with HIIT and HV-SIT with LV-HIIT demonstrated improvements in both body composition and cardiovascular health, suggesting that various high-intensity interval modalities can confer benefits for youth populations (Lau et al., 2015; González-Gálvez et al., 2024). Importantly, both studies highlighted the need to enhance children’s and adolescents’ positive attitudes toward exercise, emphasizing that individuals are more likely to adopt and sustain healthy behaviors when perceived benefits outweigh perceived barriers. Based on the current findings, time-efficient high-intensity interval training with shorter recovery intervals appears to offer a more tolerable and cost-effective approach for maintaining or reducing bodyweight among overweight and obese children and adolescents (Lau et al., 2015; Weinberg and Gould, 2023).

### 4.4 Practical implications

The present study defined LV-HIIT as a single exercise session with a total duration of no more than 30 min (including warm-up, inter-set recovery, and cool-down phases), in which the total time spent in vigorous activity did not exceed 10 min. This definition ensured that the intervention maintained the characteristics of low-volume training while addressing the issues of time efficiency and safety for children and adolescents. The included studies incorporated various exercise modalities, such as outdoor running, cycling, and full-body bodyweight training. These formats align well with everyday activity settings, thereby enhancing the practicality and feasibility of LV-HIIT for children and adolescents.

In addition, previous studies have explored the implementation of HIIT within school settings (Duncombe et al., 2022; Liu et al., 2024); however, several challenges remain in applying such interventions effectively in this context. First, ensuring the authenticity of intensity control is a major concern: outside the laboratory environment, it is difficult to guarantee that students actually reach the target intensity. Monitoring tools include heart rate monitors, accelerometers (for assessing vigorous physical activity, VPA), and the rating of perceived exertion (RPE) scale are not easily deployed in real-world exercise scenarios when supervisors are absent. Therefore, when objective monitoring tools are unavailable, self-reported measures (e.g., RPE or exercise logs) may offer a practical means to enhance implementation feasibility. Nevertheless, because of their inherent subjectivity, future studies should validate these tools or combine them with objective indicators to ensure intervention fidelity and accurate interpretation of outcomes. Second, the lack of process evaluation in randomized controlled trials (RCT) often results in insufficient documentation and interpretation of intervention fidelity, adherence, and contextual factors. Third, participant attrition remains a common issue, with reasons such as absence, illness, limited time availability, and school transfers. Therefore, implementing exercise interventions effectively in school environments requires careful consideration of curriculum alignment during the study design phase to avoid disrupting normal educational objectives. Moreover, appropriate motivational strategies, flexible intervention schedules, and adequate sample sizes should be ensured to reduce high dropout rates. It is noteworthy that LV-HIIT, combining time efficiency with demonstrated benefits in improving body composition and cardiovascular health, has potential to be integrated into recess activities or physical education classes, providing a practical strategy to enhance physical activity levels among children and adolescents. Furthermore, the emerging concept of exercise snacking may further facilitate real-world application. It is defined as any exercise pattern, regardless of intensity, accumulated in either continuous or intermittent bouts lasting ≤10 min (including multiple intermittent sets), performed several times per day (≥2 times/day), with intervals between bouts allowing for full recovery or lasting ≥30 min (Yin et al., 2025; Yin et al., 2024a; Yin et al., 2024b; Yin et al., 2024c; Yin et al., 2024d). Consequently, LV-HIIT protocols could feasibly be implemented multiple times per day to increase physical activity levels among children and adolescents.

Moreover, integrating AI-assisted tools into such short, high-frequency exercise formats may further enhance their feasibility in school environments. AI-based systems (e.g., automated intensity detection, real-time feedback platforms, and motivational prompt generators) can help ensure training quality and support students’ engagement without requiring continuous instructor supervision. However, recent evidence suggests that excessive or poorly regulated AI chatbot use may relate to adverse psychological outcomes in youth populations. Therefore, the application of AI within school-based exercise programs should be carefully designed, incorporating appropriate usage limits, human oversight, and monitoring of students’ psychological responses to ensure both physical and mental health benefits (Zhang et al., 2025).

### 4.5 Limitations

In addition, several limitations should be acknowledged. First, although this meta-analysis included 23 studies, the possibility of missing some unpublished or gray literature cannot be ruled out. Therefore, there is a potential risk of publication bias and incomplete results. Nevertheless, we conducted a comprehensive search across major databases and performed both funnel plot inspection and Egger’s test to minimize and verify the presence of such bias. Second, only nine studies directly compared LV-HIIT and MICT, which limits our ability to interpret the findings and identify potential moderators. Future research should include more high-quality trials to strengthen the evidence base. Similarly, in the comparison between LV-HIIT and control groups, only a limited number of moderator and dose-response analyses could be conducted due to the small number of studies, restricting our understanding of heterogeneity sources. More studies are therefore needed to further investigate the effects of LV-HIIT in children and adolescents. Third, a notable proportion of the included studies were rated as having moderate to high risk of bias, mainly in the domains of outcome measurement and selective reporting. Specifically, most studies lacked blinding of outcome assessment and did not preregister their study protocols on public data platforms. Moreover, according to the GRADE assessment, the overall quality of evidence for most outcomes was rated as low, indicating the need for future researchers to adhere more rigorously to experimental standards and improve methodological quality. In addition, only 246 of the participants included in this meta-analysis were female. The limited number of female participants prevented us from conducting sex-specific subgroup analyses, thereby hindering our ability to examine potential sex differences in the effects of LV-HIIT and partially restricting the generalizability of the findings across genders. Moreover, socioeconomic status was not considered as a key demographic factor during the study design and data extraction stages, which further limits the interpretation and generalization of the results. Future research should systematically collect and report socioeconomic information to enhance the representativeness and external validity of the evidence. Finally, we did not conduct subgroup analyses based on intensity metrics due to the heterogeneity in how intensity was quantified across studies. Several intensity categories contained only one or two studies, and the physiological relationships among VO_2_max, HRmax, and speed/power-based indicators are not strictly equivalent. Conducting subgroup analyses under these conditions would have been statistically underpowered and potentially misleading; therefore, intensity information was presented descriptively rather than used for stratified analyses. Considering that the trim-and-fill method may yield unstable or overcorrected results when the number of included studies is small or heterogeneity is high, we did not apply this method to adjust for publication bias. Therefore, the interpretation and generalization of our findings should be approached with caution.

## 5 Conclusion

This meta-analysis demonstrated that LV-HIIT effectively improves body composition and cardiovascular health among children and adolescents. When compared with MICT, both interventions produced comparable outcomes; however, LV-HIIT showed greater time efficiency. Subgroup analyses indicated that weight status, age, and intervention duration may serve as key moderators influencing the effects of LV-HIIT on bodyweight, SBP, DBP, and VO_2_max. The dose-response analysis further revealed that longer intervention durations, higher training frequencies, and a greater number of repetitions were correlated with reductions in blood pressure, whereas extending the duration of a single high-intensity repetition appeared to attenuate this effect. Descriptive findings additionally showed that the improvements in body composition and cardiovascular health induced by LV-HIIT were comparable to those achieved by SSG and HV-SIT, but more pronounced than those observed with MIIT. It should be noted that, given the limited number of included studies and potential biases, these results should be interpreted with caution. Nonetheless, the present findings provide valuable evidence to inform exercise prescription development for children and adolescents, particularly in contexts emphasizing a balance between time efficiency and health benefits.

## Data Availability

The original contributions presented in the study are included in the article/Supplementary Material, further inquiries can be directed to the corresponding author.
