# Supplementary figures and images for: Is low-volume high intensity interval training a time-efficient strategy for improving body composition and cardiovascular health in children and adolescents? Evidence from a systematic review and three-level meta-analysis
Source: Front Physiol. 2025 Dec 19;16:1736441. doi: 10.3389/fphys.2025.1736441 (PMC12757267; doi:10.3389/fphys.2025.1736441)

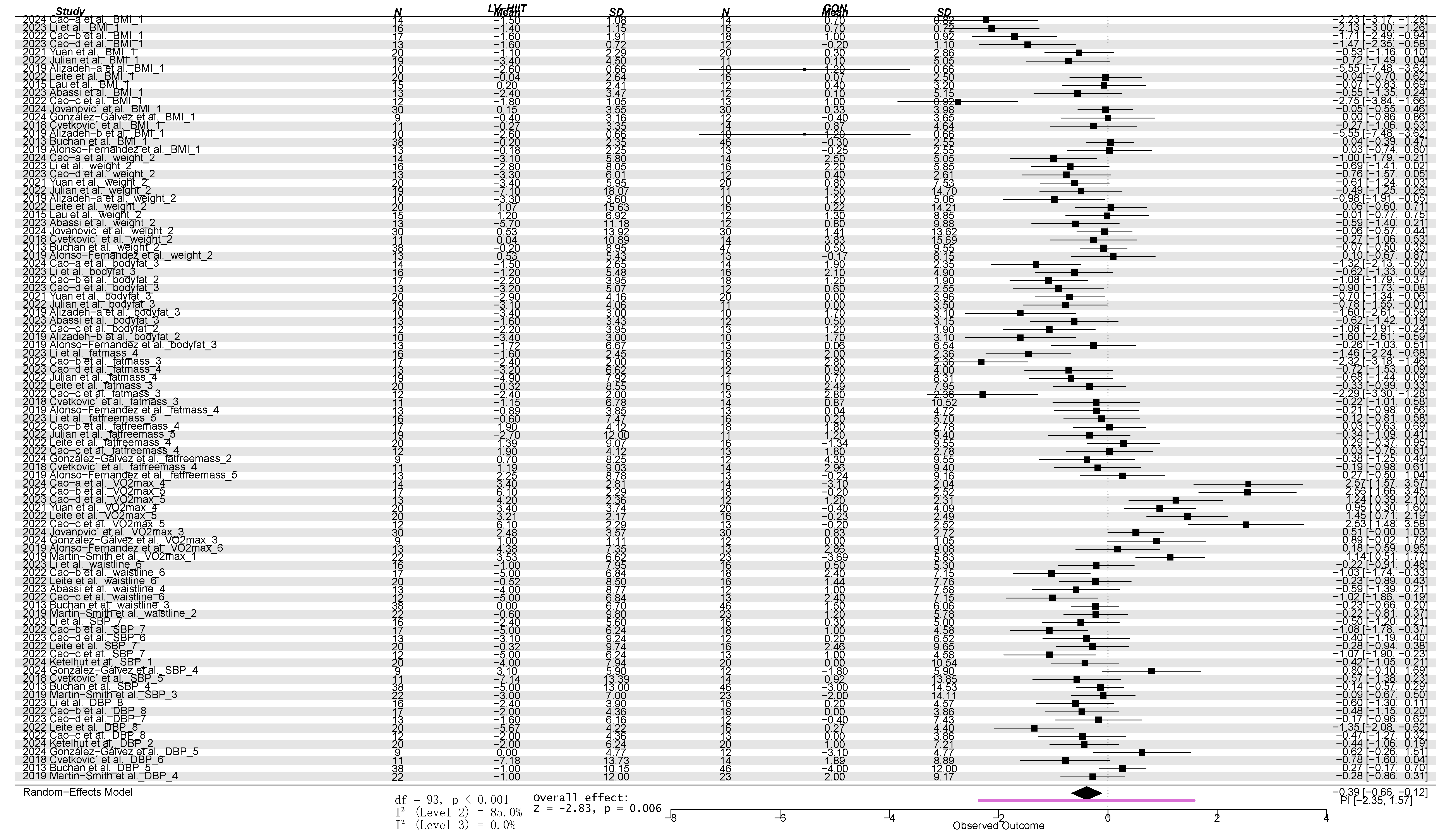

Supplement: Supplementary file 1 [file DataSheet1.zip › All Electronic Supplementary Material/supple figure/Supplementary S2.jpg]

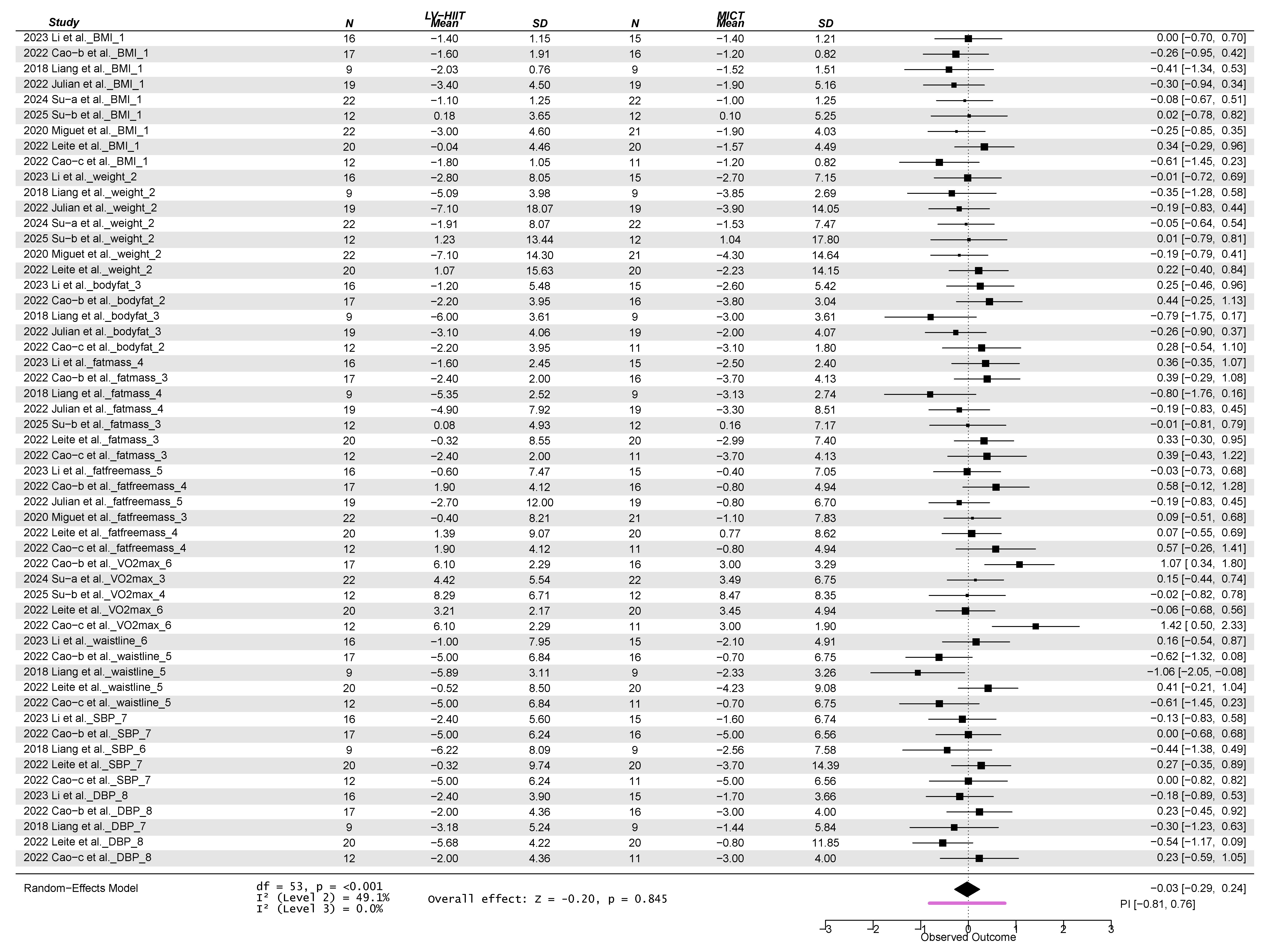

Supplement: Supplementary file 1 [file DataSheet1.zip › All Electronic Supplementary Material/supple figure/Supplementary S4.jpg]

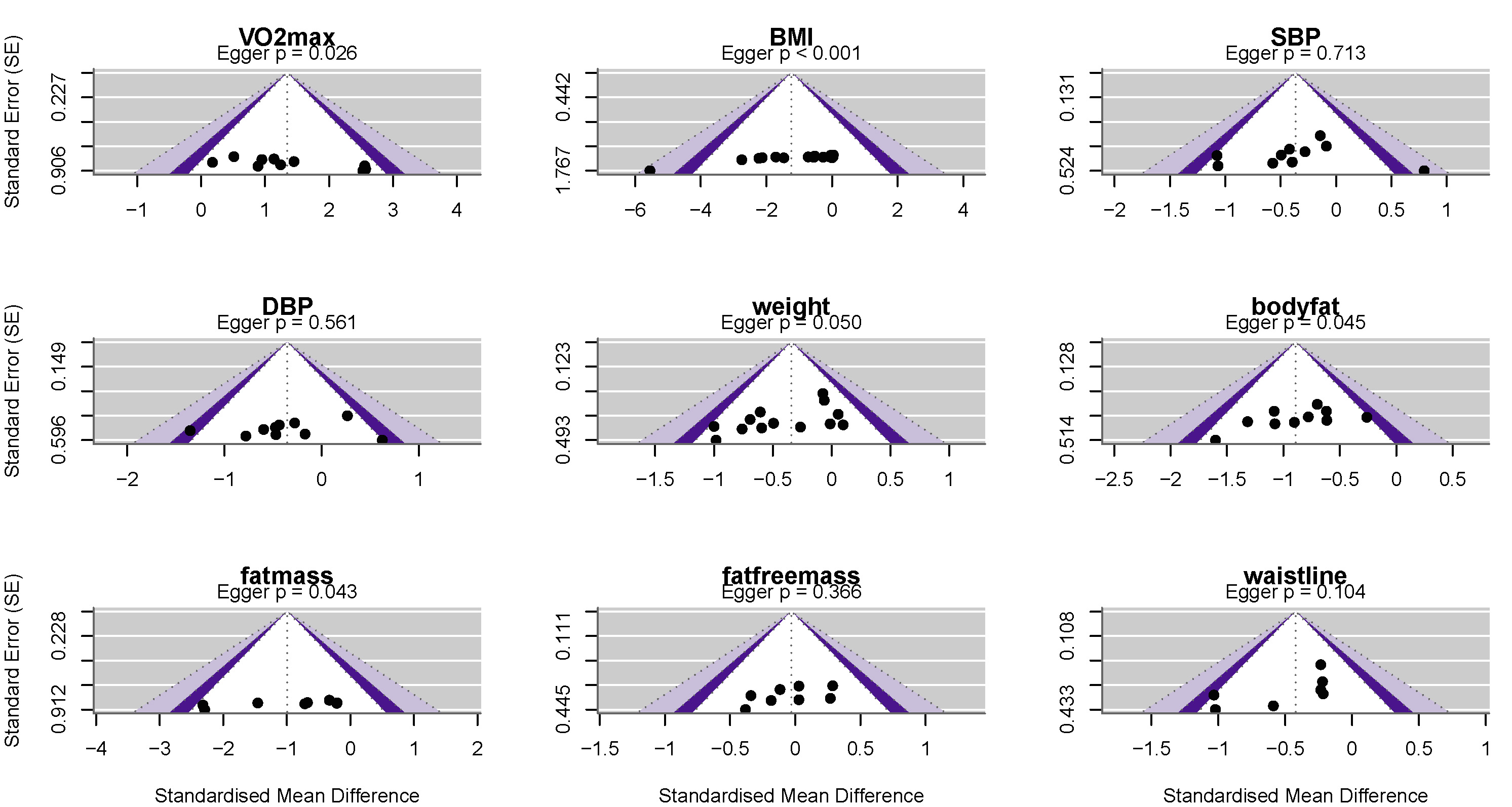

Supplement: Supplementary file 1 [file DataSheet1.zip › All Electronic Supplementary Material/supple figure/Supplementary S7.jpg]

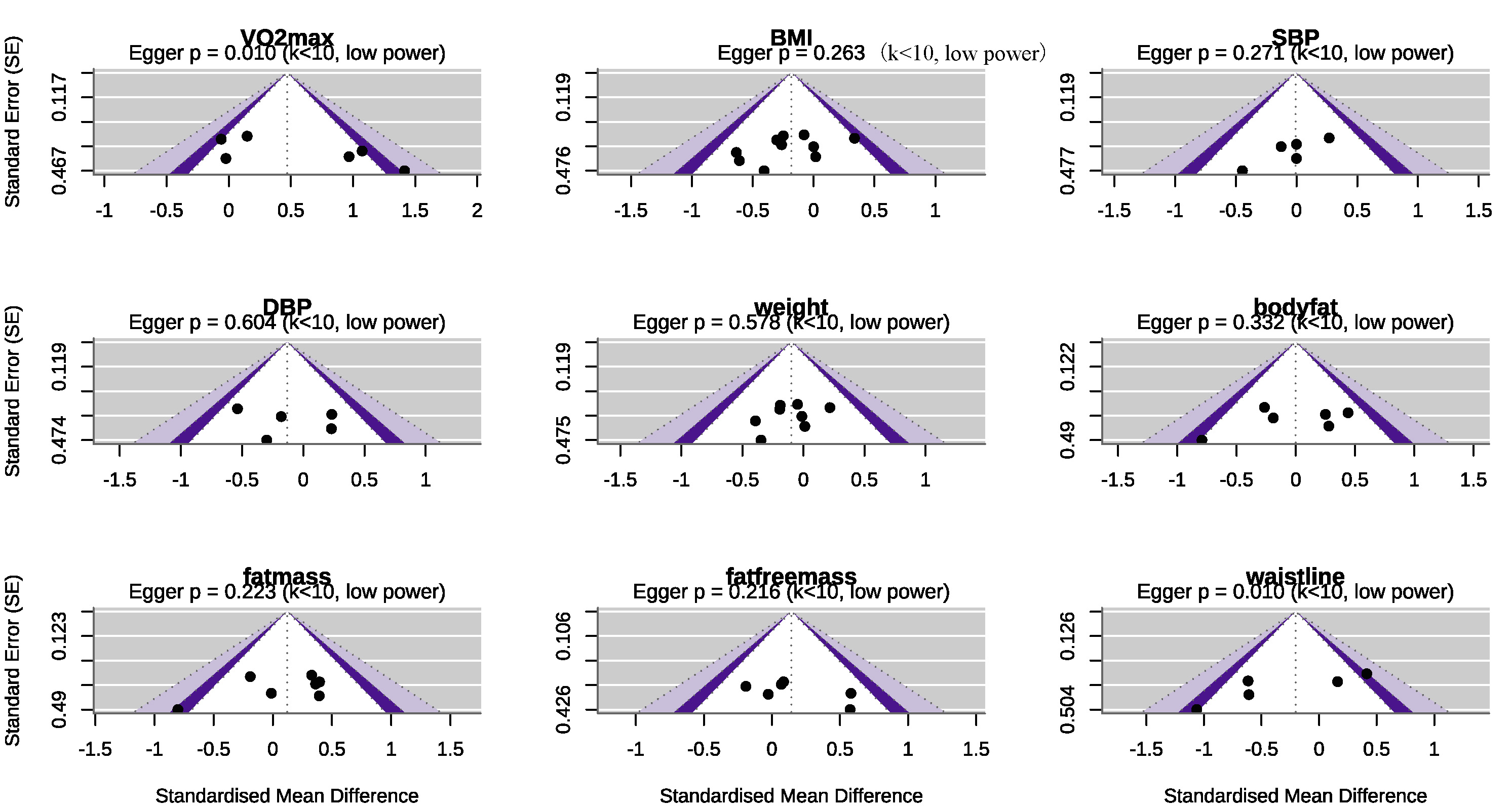

Supplement: Supplementary file 1 [file DataSheet1.zip › All Electronic Supplementary Material/supple figure/Supplementary S8.jpg]
